# The Strong Protective Action of Ce^3+^/F^−^ Combined Treatment on Tooth Enamel and Epithelial Cells

**DOI:** 10.3390/nano12173034

**Published:** 2022-09-01

**Authors:** Anton L. Popov, Nadia M. Zholobak, Alexander B. Shcherbakov, Taisiya O. Kozlova, Danil D. Kolmanovich, Artem M. Ermakov, Nelli R. Popova, Nikita N. Chukavin, Ernest A. Bazikyan, Vladimir K. Ivanov

**Affiliations:** 1 Kurnakov Institute of General and Inorganic Chemistry of the Russian Academy of Sciences, Moscow 119991, Russia; 2 Institute of Theoretical and Experimental Biophysics, Russian Academy of Sciences, Pushchino 142290, Moscow Region, Russia; 3 Zabolotny Institute of Microbiology and Virology, National Academy of Sciences of Ukraine, Kyiv 03680, Ukraine; 4 Moscow Region State University, Moscow 141014, Russia; 5 Yevdokimov Moscow State University of Medicine and Dentistry, Moscow 127473, Russia

**Keywords:** ceria, nanoparticles, enamel, tooth, dental pulp stem cells, dental diseases

## Abstract

We studied the toxic effects of cerium and fluoride species on human dental pulp stem cells and epithelial cells of *Cercopithecus aethiops* as a surrogate for the human oral mucosa. The sequential use of CeCl_3_ and NH_4_F solutions in equimolar sub-toxic concentrations enabled the possible toxic effects of individual components to be avoided, ensuring the preservation of the metabolic activity of the cells due to the formation of CeF_3_ nanoparticles. Cerium fluoride nanoparticles and terbium-doped cerium fluoride nanoparticles exhibited neither cytotoxicity nor genotoxicity to dental pulp stem cells, even at high concentrations (10^−4^ M). In millimolar concentrations (from 10^−5^–10^−6^ M), these nanoparticles significantly increased the expression of genes responsible for the cell cycle, differentiation and proliferation. The formation of cerium fluoride on the surface of the mucous membrane and teeth provided protection against the development of carious lesions, periodontitis, ROS attacks and other inflammatory diseases of the oral cavity. Luminescent CeF_3_: Tb nanoparticles enabled the visualization of tooth enamel microcracks.

## 1. Introduction

Caries is a dental disease that occurs with the destruction of hard tooth tissues, generally in acidic erosion conditions. The inorganic part of tooth hard tissue consists mainly of hydroxyapatite crystalline species (calcium phosphates). In the crystal lattice of hydroxyapatite, hydroxyl ions can be substituted by fluoride ions, thus, forming fluorapatite, which possesses enhanced microhardness and demonstrates higher resistance to the action of acids formed in the oral cavity. 

The beneficial effects of fluoride on human oral health are well-known; fluorides have a protective effect against both dental hard tissue erosion and caries formation through demineralization inhibition and remineralization promotion. Fluoride ions can also protect against fermentation attack by *Streptococcus sobrinus* artificial plaque on tooth enamel [1]; however, an excess of fluoride ions has several adverse effects [2,3]. Exposure to high fluoride doses has been reported to induce apoptotic cell death in ameloblasts, odontoblasts and osteoblasts. 

Tabuchi et al. studied the effects of fluoride ions on rat oral epithelial cells and observed increased cell death with an increasing concentration of NaF from 2 to 4 mM [4]. The induction of apoptosis by this compound was confirmed by the activation of caspase-3 with a concomitant increase in the expression of *Atf3*, *Ddit3* and *Fos* genes. The differentially expressed genes are likely to be involved in cell death accompanying endoplasmic reticulum (*ER*) stress. 

A recent study investigating the effects of fluoride ions on the proliferation of cells and gene expression showed that fluoride exposure increased human B lymphocytes’ viability at relatively low levels (10–160 µM); however, when the concentration reached 320 µM, cell proliferation was significantly inhibited. Fluoride ions induce apoptosis in mammalian cells, both in vitro and in vivo [5]. At a concentration of 50 μM, fluoride ions are able to increase the production of superoxide anion radicals in the mitochondria of osteoblasts, while reducing their oxygen consumption [6]. 

Increased production of reactive oxygen species (ROS) occurs due to oxidant system suppression: superoxide dismutase and catalase dramatically reduce their activity upon fluoride intoxication. At the same time, insoluble cerium fluoride is capable of protecting cells from oxidative stress [7] and accelerating mitogenesis via neoblastic activation [8].

Cerium species are promising for biomedical practice [9], including dental care, e.g., in ceria-containing orthodontic polymers [10]. Cerium oxide nanoparticles protect human dental stem cells from oxidative insult [11]. Recently, cerium oxide particles were successfully used as a filler to tune the radiopacity of dental adhesives [12]. Soluble and insoluble cerium compounds possess notable antibacterial activity [13] and appear to have a high potential for use against dental bacteria (including *Streptococcus mutans* [14]) that are responsible for the cariogenic process. 

In their experiments with four common bacteria (i.e., *E*. *coli*, *S*. *aureus*, *B*. *subtilis* and *B*. *cereus*), Anastasiou et al. showed that doping with Ce^3+^ enhanced the regenerative potential of dental pulp stem cells, while retaining the good antibacterial profile of fluorapatite [15]. The incorporation of cerium ions into dental enamel and the formation of cerium-apatite has been reported [16]. Wegehaupt et al. established that cerium chloride is able to significantly reduce the acid susceptibility of dentine [17] and demonstrated the anti-erosive potential of the combination of fluoride ions, cerium chloride and laser irradiation on dentine [18]. 

Cerium chloride and its combination with fluoride species can significantly reduce carious mineral loss and the progression of lesion depth [19]. Zhang et al. showed that the combination of fluoride and cerium ions was more efficient in the prevention of root surface caries than lanthanide ions alone [20]. 

Later, Jaisingh et al. demonstrated that cerium chloride reduced demineralization, and improved remineralization, for artificial caries lesions in human enamel in vitro when only subjected to pH cycling [21]. Lanthanum, cerium and fluoride species (500 ppm) were shown to provide demineralization resistance in an acid resistance and remineralization study on human root surface [22]. Jin et al. demonstrated the beneficial effect of lanthanum, cerium and fluoride species on the prevention of root surface caries [23].

Soluble calcium species are well-known fluoride neutralizers; they reduce the fluoride bioavailability in lethal models of fluoride poisoning [2,24,25]. The mechanism of their protective action is thought to be the formation of insoluble calcium fluoride salts, which results in binding and inactivating the fluoride ions. The solubility constant of CeF_3_ (K_sp_ = 8 × 10^–16^) is substantially lower than that of fluorspar, CaF_2_ (K_sp_ = 4 × 10^–11^) [7]; this leads to the expectation of even more effective protection against fluoride ions when using cerium soluble salts. 

The current study features the toxic effects of cerium and fluoride species on human dental pulp stem cells (DPSc) and epithelial cells of *Cercopithecus aethiops* as a surrogate for the human oral mucosa. The study also analyses the protective effect of CeF_3_:Tb nanoparticles in enamel defect zones and includes a comprehensive analysis of cell metabolic activity and gene expression. An analysis was made of the possible benefits of the combined application of cerium and fluoride species in dental healthcare.

## 2. Materials and Methods

### 2.1. Cell Cultures, Dental Samples, Chemicals

Cerium(III) chloride heptahydrate, terbium(III) chloride hexahydrate, calcium chloride, ammonium fluoride, hydrofluoric acid, lactic acid, sodium lactate and Arsenazo III were purchased from Sigma-Aldrich (St. Louis, MO, USA). Imidazole buffer (Glyoxaline solution) was purchased from Merck (Darmstadt, Germany). Cerium fluoride nanoparticles (both bare and terbium-doped) and cerium oxide nanoparticles were synthesized according to protocols reported elsewhere [7].

Epithelial cells MA-104 (ATCC^®^ CRL-2378) isolated from an African green monkey fetal kidney were obtained from the biobank of the Zabolotny Institute of Microbiology and Virology. Dental pulp stem cells (DPSc) were isolated from the tooth pulp of a healthy donor according to orthodontic prescription and with the informed consent of the patient. All procedures were performed in accordance with the approved clinical rules for biomaterial sampling. 

The cells were cultivated in a DMEM/F12 (1:1) medium containing 10% of fetal bovine serum (FBS), 50 μg/mL of penicillin, 50 μg/mL of streptomycin and 3 mg/mL of L-glutamine, at 37 °C in a humid atmosphere with 5% CO_2_. The cells were seeded at a density of 2 × 10^4^–2.5 × 10^4^ per cm^2^. Six hours after cell attachment, cerium salt solutions or ceria nanoparticles were added, in different concentrations (10^−3^–10^−7^ M). In the control experiments, the cells were cultured without cerium salt solutions or ceria nanoparticles.

Extracted for orthodontic reasons, human molars with sound enamel and approximately equal habit and weight (2.0 ± 0.4 g) were used; teeth were stored in a 0.5% formaldehyde solution adjusted to pH 7.5 with sodium hydroxide.

### 2.2. Visualization of Cerium and Fluoride Species Absorption and Confirmation of Cerium Fluoride Formation

For the verification of lanthanide and fluoride species adsorption on the tooth surface, human molars with sound enamel were used. To simulate a caries-like defect, part of the enamel was chipped out mechanically. We dissolved 0.425 mmol of cerium chloride heptahydrate (158.5 mg, MW = 372.58) and 0.075 mmol of terbium chloride hexahydrate (28 mg, MW = 373.38) in 5 mL of water (Ce:Tb = 0.85:0.15 mol) and named solution #1. Separately, 1.5 mmol of ammonium fluoride (55.5 mg, MW = 37) was dissolved in 5 mL of water and named solution #2. 

Each molar was immersed in lanthanide chloride solution, exposed for 5 min and immersed in ammonium fluoride solution, then rinsed with distilled water and dried. Tooth luminescence was studied under irradiation, using a Philips TUV G15 T8 (Philips, Hamburg, Germany) germicidal quartz lamp with a maximum emission at 253.7 nm. For the independent confirmation of CeF_3_:Tb nanoparticles formation, the following experiment was performed. A few drops (~0.5 mL) of saliva were added to solution #2. Solution #1 was quickly added to solution #2 under agitation. The terbium-doped cerium fluoride nanoparticles formed were studied by luminescent spectrophotometry using a QE-65000 spectrometer, Ocean Optics (Orlando, FL, USA) and TEM microscopy (Leo912 AB Omega, Carl Zeiss, Oberkochen, Germany).

### 2.3. The Cytotoxicity of the Cerium Ions and Fluoride Ions In Vitro

The cytotoxicity of the substances was analysed using the colorimetric MTT assay; the test protocol for cytotoxicity evaluation was adopted from elsewhere [26]. Briefly, different concentrations of the substances (10 μM–2 mM) were added and cells were incubated for 24 h, at 37 °C in humid air (98%) containing 5% CO_2_. Four hours prior to the end of the exposure period, the supernatant was removed, and MTT (3-(4,5-Dimethyl-2-thiazolyl)-2,5-diphenyl-2H-tetrazolium bromide, Sigma-Aldrich, St. Louis, MO, USA, #M5655) solution in PBS (0.1 mg/mL, 100 μL/well) was added to the cells. 

Upon the completion of the exposure period, the supernatant was removed, and a lysis solution containing 0.1% SDS (Sigma-Aldrich, #L3771) solution in DMSO (Sigma-Aldrich, #D8418) was added. Plates were shaken for 5 min, placed on a Multiskan MS Microplate Reader (Thermo Labsystems, Santa Rosa, CA, USA), and the absorbance was read colorimetrically at 492 nm. Each experiment was repeated three times, with four replications.

### 2.4. The Protective Effect of Cerium or Calcium Species against Fluoride Ions In Vitro

The protective effect of cerium or calcium species was studied according to therapeutic, and prophylactic, schemes. Briefly, in the prophylactic scheme, solutions of cerium chloride or calcium chloride, of different concentrations (10 μM–2 mM), were added to the cell monolayers; 24 h later, a solution of ammonium fluoride was added to the cells. The concentration of fluoride ions in each well was 0.5 mM, corresponding to 50% viability in the acute toxicity test. The cells were incubated in fluoride media for 4 h and subsequently washed and stained with crystal violet. 

The excess dye was then removed and the stained monolayer was washed with distilled water and dried. The absorbance of stained cells was measured at 492 nm using a Thermo/LabSystems Multiskan MS Microplate Reader. The optical density of stained cells corresponded to their viability. The percentage of the cells absorbing crystal violet was determined according to the formula $\frac{D_{ex}}{D_{contr}}\cdot 100$, where *D_ex_* is the optical density of the experimental wells and *D_contr_* is the optical density of the intact (control) wells. 

Statistical analysis of the data obtained was performed using BioStat 2009 Professional 5.8.1 (Analystsoft, Walnut, CA, USA) software, in accordance with standard recommendations. Experimental data were presented in terms of the median and interquartile range, Me (LQ-UQ), where Me = median (50% percentile), LQ = 25% percentile and UQ = 75% percentile. For the therapeutic scheme, the sequence of cerium/calcium and fluoride species addition was reversed. Each series of experiments was repeated five times.

### 2.5. The Protective Effect of Cerium-Containing Species against ROS In Vitro

The protective action of cerium ions and ceria nanoparticles against ROS was determined as described elsewhere [7]. Briefly, the solutions of tested species (0.005–0.3 mM) were introduced to the cell monolayers, and 18 h later hydrogen peroxide was added to the cells, (the H_2_O_2_ concentration in each well was 0.5 mM, corresponding to 5% viability in the toxicity test). The cells were incubated for 4 h under conditions of oxidative stress, then washed and stained with crystal violet. Subsequent protective effect analysis and data processing were essentially the same as in the previous series of experiments (see above, “The protective effect of cerium or calcium species against fluoride ions in vitro”). Each series of experiments was repeated five times.

### 2.6. Live/Dead Assay

A Live/Dead Viability Kit (Invitrogen, Thermo Fischer, Carlsbad, CA, USA) was used to evaluate the cytotoxic effects of cerium-containing nanoparticles. The kit contained two fluorescent dyes for selective cell labelling. The green signal (SYTO 9 dye, λ = 485/498 nm) characterized the live cells, and the red signal (propidium iodide dye, λ = 535/617 nm) characterized the dead cells. Cells were seeded into 96-well plates and the kit was used according to the manufacturer’s protocol. Cells were visualized and photographed 25 min after adding the dyes using an Axiovert 200 fluorescent microscope (Carl Zeiss, Oberkochen, Germany) and a Canon A620 digital camera (Canon, Tokyo, Japan). For each cell group, four fields in each well were examined.

### 2.7. Analysis of Mitochondrial Membrane Potential (MMP)

Mitochondrial membrane potential (MMP) was determined by TMRE (tetramethylrhodamine, ethyl ester, ThermoFisher, Carlsbad, CA, USA) dye using fluorescence microscopy. TMRE accumulates in the mitochondrial membrane in a potential-dependent manner. 

Cells were seeded into 96-well tissue culture plates (Greiner, Kremsmünster, Austria) at a density of 5 × 10^4^ cells/well and cultured in a CO_2_ incubator at 37 °C for 24, 48 and 72 h, with different concentrations of nanoparticles. The cells were preincubated with 1 μM TMRE in the HBSS in a CO_2_ incubator at 37 °C for 30 min. Next, the cells were washed twice by HBSS and analysed using an Axiovert 200M (Carl Zeiss, Oberkochen, Germany) inverted fluorescence microscope (Carl Zeiss, Germany) at 200× magnification.

### 2.8. Real-Time PCR

A kit for mRNA isolation with magnetic particles was used, according to the manufacturer’s protocol (Sileks, Moscow, Russia). Reverse transcription was performed using a kit supplied by Sileks (Russia), using oligo(dT) primer, following the manufacturer’s protocol. The cDNA derived was used as a template for real-time PCR. The reaction was conducted using a mixture with SybrGreen (Syntol, Moscow, Russia), using a CFX-96 thermal cycler (BioRad, Hercules, CA, USA) or ABI 7500 Fast Real-Time PCR System (Thermo Fisher Scientific, Waltham, MA, USA). The expression level of 96 genes, responsible for 25 key cellular processes, was estimated (Table 1). The analysed genes were selected using the database http://www.sabiosciences.com/ (accessed on 1 April 2022) for PCR profiling of different biological processes. 

The transcription level was normalized using the expression of housekeeping genes encoding β-actin, RPLP0 (ribosomal protein, large, P0) and GAPDH (glyceraldehyde-3-phosphate dehydrogenase). Gene-specific primers were picked using Primer Express software (Applied Biosystems, USA). Each measurement was made with two replications (internal replication) and averaged for two independent samples. Samples without reverse transcription were used as a control. Analysis of the expression data was performed using online services at http://www.sabiosciences.com/ (accessed on 1 April 2022), mayday-2.14 software (Center for Bioinformatics, Tübingen, Germany) (accessed on 15 April 2022) and Genesis software.

### 2.9. Assessment of Tooth Enamel Protection

For the evaluation of tooth enamel protection against acidic dissolution by cerium- and fluoride-containing species, human molars were used. Twelve teeth with sound enamel, of approximately equal weight (2.0 ± 0.2 g) and habitus, were divided into four groups: nontreated (control); treated with 10 mM cerium chloride; treated with 10 mM ammonium fluoride; and treated with 10 mM cerium chloride and then by 10 mM ammonium fluoride. 

An etching lactate buffer (5% *w*/*v*) was prepared using the solution containing 0.4510 M lactic acid and 0.1041 M sodium lactate, adjusted to pH 3.0 with sodium hydroxide. The release of calcium ions was measured using the colorimetric method, with Arsenazo III indicator [27]. The reagent used for the determination of calcium ions was composed of 100 mM imidazole buffer (pH 6.5) and 0.12 mM Arsenazo III. The absorption was determined at λ = 650 nm. 

Each molar was immersed in the etching buffer solution (5 mL in glass tube) to the level of the enamel zone and exposed for 1 min; after 1 min of exposure, each molar was transferred to the next tube containing a fresh lactate buffer, and the remaining supernatant solution used was analysed. The total erosive attack time for each molar was 10 min. The square of enamel immersed was calculated for every molar, considering the tooth habit.

### 2.10. Statistical Data Processing

The results obtained were processed using statistical criteria. Variation statistics were used to estimate the significance of the results. Any significant difference from the control was assessed using the nonparametric Mann–Whitney U test.

## 3. Results

### 3.1. Toxicity and Cellular Metabolic Activity

The use of cerium and fluoride compounds in human dental healthcare is directly related to their effect on the oral mucosa; therefore, monkey epithelial cells MA-104 (having the maximum species proximity to humans) were selected for cytotoxicity and metabolic activity studies. Ce^3+^ and Ca^2+^ ions are close in terms of their biochemical properties [28]; thus, calcium salt solution was used as a reference. Equimolar toxic concentrations of cerium or calcium salts as well as 10-fold higher concentrations of salts were used.

**Figure 1:**
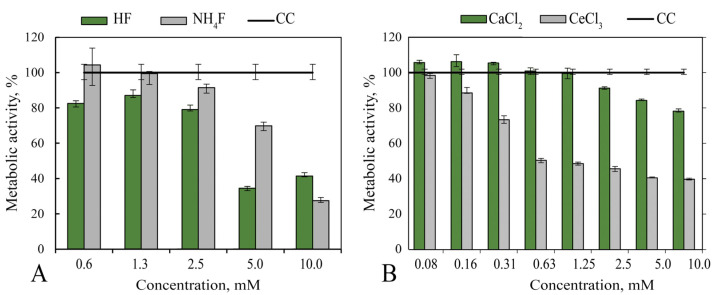
Metabolic activity (MTT test) of MA-104 cells 2 h after the addition of various concentrations of: (**A**) HF and NH_4_F and (**B**) calcium chloride and cerium chloride. CC—cell control, 100%.

The 24-h contact of MA-104 cells with NH_4_F solution was accompanied by 60–70% inhibition of their metabolism (MTT test), CC50 = 7.0 mM, CC0 = 1.3 mM (Figure 1A). For hydrofluoric acid, CC50 = 3.0 mM, CC0 was not determined. According to the results obtained on cytotoxicity, a 10 mM NH_4_F concentration was considered as toxic in further experiments in vitro.

Studies of the metabolic activity of MA-104 cells in the presence of cerium and calcium salts showed that calcium chloride had low toxicity (CC0 = 1.0 mM), while cerium chloride was much more toxic (CC0 = 0.1 mM) (Figure 1B). In the presence of calcium chloride, no changes in metabolic activity of the cells were observed; for cerium chloride, there was a short period of transition from non-toxic behaviour to metabolism-inhibiting action: a five-fold increase in salt concentration was accompanied by a 50% decrease in cell metabolism (CC50 = 0.5 mM). For calcium chloride, the calculated CC50 value was about 50 mM, i.e., 100-times higher.

**Figure 2:**
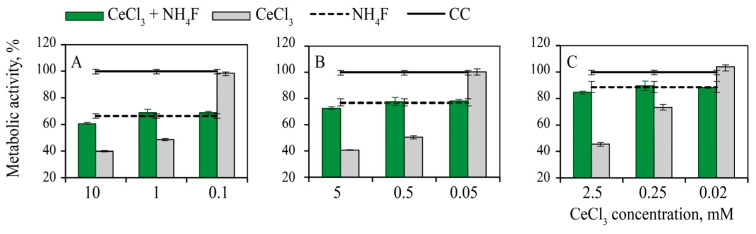
Metabolic activity of MA-104 cells pre-treated with cerium chloride 5 min before the addition of ammonium fluoride: (**A**) 10 mM, (**B**) 5 mM and (**C**) 2.5 mM. CC—cell control.

The inhibition of cell metabolism caused by cerium chloride addition was found to decrease significantly after subsequent treatment with equimolar concentrations of NH_4_F (Figure 2). The same effect was observed when the NH_4_F concentration was an order of magnitude higher than the concentration of CeCl_3_. Thus, the addition of equimolar, or even significantly higher, concentrations of NH_4_F to the cells, treated with a CeCl_3_ solution in toxic concentrations, cancelled the development of the toxic effects induced by Ce^3+^ ions. The metabolic activity of the cells treated sequentially with cerium chloride and ammonium fluoride slightly differed from the metabolic activity of the cells treated solely with NH_4_F. Interestingly, non-toxic CeCl_3_ concentrations had no effect on the metabolic activity of cells subsequently treated with NH_4_F.

**Figure 3:**
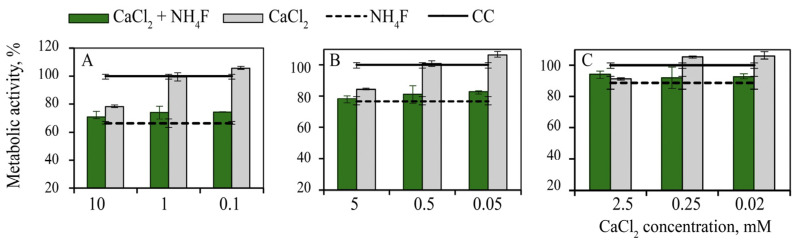
Metabolic activity of MA-104 cells pre-treated with calcium chloride 5 min before the addition of ammonium fluoride: (**A**) 10 mM, (**B**) 5 mM and (**C**) 2.5 mM. CC—cell control.

Ca ions in the concentration range of 0.025–0.25 mM caused a small, but statistically significant, increase in cell metabolic activity, while 2.5–10.0 mM Ca concentrations caused the inhibition of cell metabolism by 10–20%. Ammonium fluoride (2.5–10.0 mM) also caused a decrease in the metabolic activity of the cells, by 10–35%. Over the entire range of CaCl_2_ concentrations, the addition of NH_4_F to the cells caused an additional decrease in their metabolic activity compared with treatment with NH_4_F alone (Figure 3).

**Figure 4:**
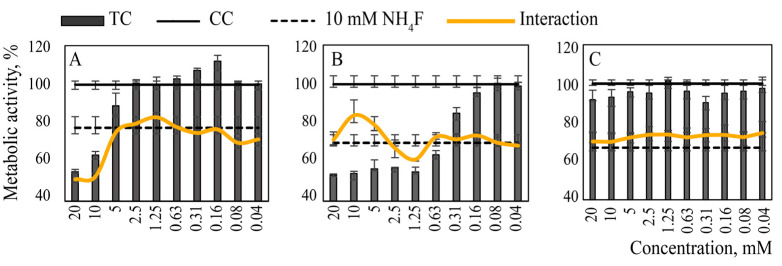
Metabolic activity of MA-104 cells pre-treated with toxic concentrations (TC) of: (**A**) cerium oxide nanoparticles, (**B**) cerium chloride and (**C**) calcium chloride. CC—cell control. “Interaction” lines demonstrate the effect of calcium or cerium species added 5 min before the addition of 10 mM NH_4_F.

Based on the results obtained, a wider concentration range of CeCl_3_ and CaCl_2_ solutions was tested in combination with a known toxic concentration of NH_4_F (10 mM), causing 30% (22–32%) inhibition of metabolic activity. The interval between the treatment of the cells with CeCl_3_ or CaCl_2_ solutions and NH_4_F solutions was set at 5 min, which could be used in practice for a mouth rinse or for a similar medical tooth treatment. A colloidal solution of citrate-stabilized cerium dioxide nanoparticles of the same concentrations was used as an additional control. The results are shown in Figure 4.

The sequential treatment of the cells with CeCl_3_ and NH_4_F solutions at equimolar concentrations ensured the preservation of cell metabolic activity; for the cells treated with 10 mM CeCl_3_ only, the metabolic activity was 55% of that of the control; for the cells treated with NH_4_F: 70%; and for the cells treated sequentially with both CeCl_3_ and NH_4_F: 80–95%. The use of CeO_2_ colloidal solution did not increase the metabolic activity of cells treated with NH_4_F. The addition of CaCl_2_ solution to the cells before their treatment with NH_4_F did not lead to any statistically significant change in cell metabolism in comparison with the cells treated with NH_4_F only.

Thus, we found that contact of monkey kidney epithelial cells MA-104 with cerium chloride solution with concentrations of 0.1 mM or higher was accompanied by a sharp concentration-dependent decrease in their metabolic activity. The addition of NH_4_F solution in an equimolar, or even a ten-times higher concentration, to the cells after 40 min of CeCl_3_ treatment eliminated the adverse effects of cerium species. The ex tempore (5 min after treatment) addition of an equimolar NH_4_F solution also ensured good cell viability and even exceeded that of the cells treated with either CeCl_3_ only or NH_4_F only.

### 3.2. Protective Effect of Cerium and Fluoride Species against H_2_O_2_-Induced Oxidation Stress

The protective action of calcium chloride, cerium chloride, ammonium fluoride and cerium fluoride against oxidative stress caused by ROS (hydrogen peroxide) action on MA-104 cells was studied in the concentration range of 0.005–0.31 mM; these concentrations were selected as being non-toxic for MA-104 cells, according to the data presented above. Since cerium oxide nanoparticles are a well-known ROS protector [9], this material was also included in the research design as a reference; the protective action of calcium ions was evaluated for comparison, too.

**Figure 5:**
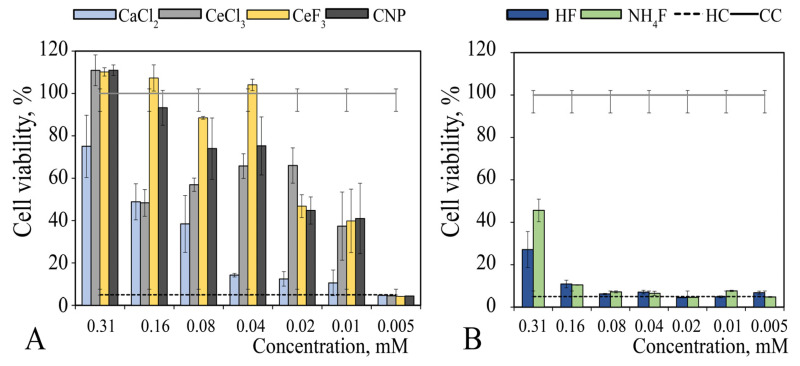
Viability of MA-104 cells under H_2_O_2_-induced oxidative stress. Cells were pre-treated with: (**A**) cerium or calcium species and (**B**) fluoride species. CNP—ceria nanoparticles, HC—hydrogen peroxide toxicity control and CC—intact cell control.

MA-104 cells were treated with cerium or calcium species for 18 h and cell viability analysis was performed (Figure 5A). 0.15–0.31 mM concentrations of calcium chloride have been shown to provide cells with some resistance to peroxide-induced oxidative stress (about 50%), while lower concentrations do not protect cells. Unlike calcium species, cerium compounds provide protection for cells at lower concentrations; in addition, the efficiency of protection is significantly higher. 

Thus, 0.31 mM cerium chloride provides 100% protection of the cells from oxidative stress induced by hydrogen peroxide; 50% and higher levels of protection were observed up to a CeCl_3_ concentration of 0.02 mM. It should be noted that CeF_3_ protects cells even more effectively than other species: a 100% level of protection was observed down to a concentration of 0.04 mM. A two-fold decrease in CeF_3_ concentration (to 0.02 mM) was accompanied by a two-fold decrease in the protective effect, although a further decrease in CeF_3_ concentration to 0.01 mM did not cause such a sharp decrease—the level of protection being 40 ± 15%. 

Terbium-doped cerium fluoride protects cells against ROS in the same manner (not shown) as a pristine CeF_3_, with some minor differences (±5–10%). The use of nanoceria was accompanied by a concentration-dependent protective action: 100% protection of the cells was observed at concentrations of 0.16–0.31 mM; the concentration range of 0.04–0.08 mM provided more than 70% protection and 0.01–0.02 mM concentrations resulted in 40% cell protection. As expected, non-toxic concentrations of HF and NH_4_F provided no protective action and did not affect the cell viability (see Figure 5B).

### 3.3. Tooth Enamel Erosion Protection and Carious Cavity Sealing by Sequential Treatment of Teeth with Cerium Chloride and Ammonium Fluoride

The end-product of bacterial glycolysis under anaerobic conditions is lactic acid; it creates acidity high enough to dissolve calcium phosphate in the tooth enamel, leading to the start of cavity formation. Clearly, acidic lactate buffer would be the best choice of surrogate oral solution for caries formation. 

**Figure 6:**
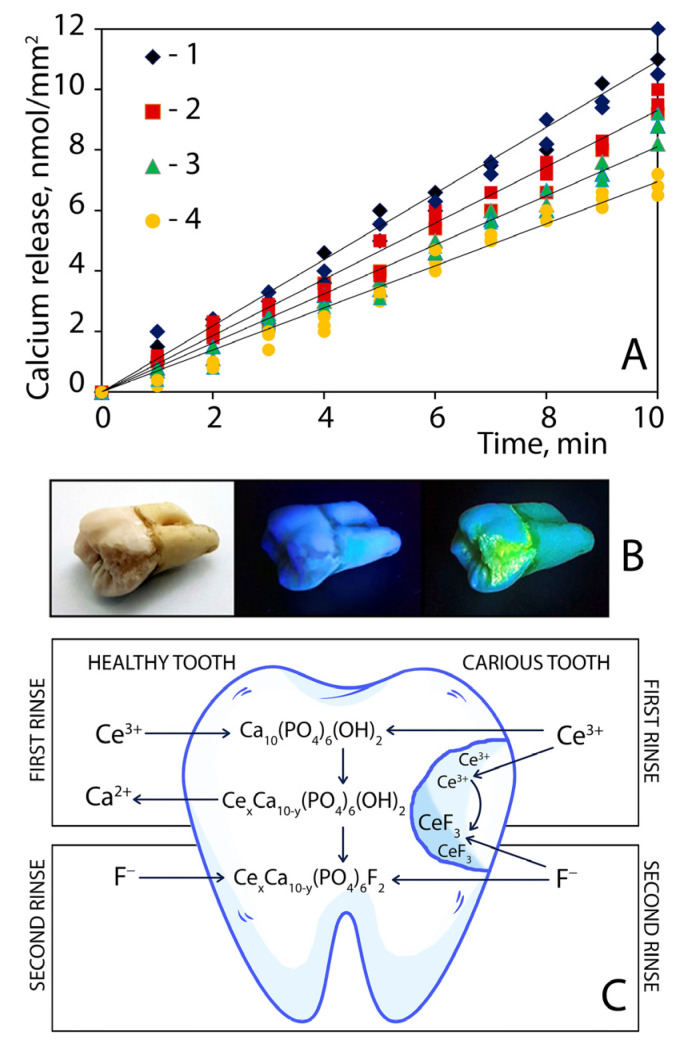
(**A**) Calcium release: 1—untreated molars (control); 2—molars treated with cerium chloride solution; 3—molars treated with ammonium fluoride solution; 4—molars treated with cerium chloride following ammonium fluoride solutions. (**B**) Molar with protective layer of CeF_3_:Tb nanoparticles in the enamel defect zones. (**C**) The proposed mechanisms of tooth protection by sequential treatment with cerium and fluoride ions.

The amount of calcium released into the lactate buffer solution during each of the ten erosive attacks for the four treatment groups of the teeth is presented in Figure 6A. The amount of calcium decreased from 11 ± 1 nmol/mm^2^ (untreated teeth) to 6.0 ± 0.6 nmol/mm^2^ (teeth treated with cerium chloride following ammonium fluoride solutions), depending on treatment conditions. Figure 6A shows that sequential treatment of teeth with cerium chloride and ammonium fluoride most significantly reduced acid corrosion of tooth enamel. The effect was much higher than in the case of using each of the components separately.

If a tooth with damaged enamel is subjected to sequential rinsing with cerium and fluoride solutions, a layer of cerium fluoride nanoparticles forms at the carious zone. This fact can be confirmed by adding a small amount of terbium ions (~15 mol%) to the cerium salt solution. CeF_3_:Tb nanoparticles formed can easily be detected due to the bright luminescence (Figure 6B), which has well-known optical characteristics (excitation and emission wavelengths). 

Moreover, the luminescence of terbium-doped cerium fluoride nanoparticles does not appear under either visible light or UVA irradiation; it can be registered under shortwave ultraviolet radiation (UVC) only and thus it does not impart the natural appearance of teeth (Figure 6B, left to right). Terbium-doped cerium fluoride nanoparticles formed in situ seem to be useful for the in vivo defectoscopy of enamel integrity and tooth surface behaviour, enabling the detection of problem areas and the prevention of caries formation.

**Figure 7:**
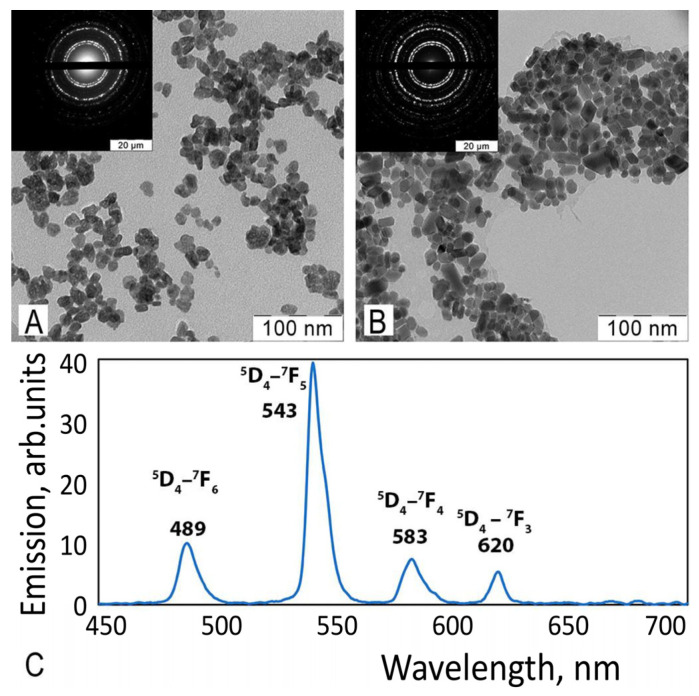
TEM images of CeF_3_:Tb^3+^ (**A**) and CeF_3_ (**B**) nanoparticles. Room temperature emission spectra of CeF_3_:Tb^3+^ (15 mol%) nanoparticles in aqueous solutions upon 254 nm excitation (**C**).

According to the SAED pattern and TEM image analyses (Figure 7), crystalline and sufficiently monodispersed (15–20 nm) CeF_3_ and CeF_3_: Tb^3+^ nanoparticles were formed upon the simple mixing of lanthanide and fluoride ions in saliva-containing media. Terbium excitation at 254 nm (characteristic 489 nm ^5^D_4_–^7^F_6_, 543 nm ^5^D_4_–^7^F_5_, 583 nm ^5^D_4_–^7^F_4_ and 620 nm ^5^D_4_–^7^F_3_ transitions) suggests energy transfer from Ce^3+^ to Tb^3+^ in the crystalline lattice; such a transfer mechanism has been thoroughly investigated for CeF_3_: Tb^3+^ nanoparticles [7].

Possible mechanisms of tooth modification by rinsing with cerium and fluoride solutions are represented in Figure 6C. Healthy enamel consists mainly of hydroxyapatite Ca_10_(PO_4_)_6_(OH)_2_. The diagram in Figure 6C (left) demonstrates the mechanism of enamel strengthening due to the sequential modification of hydroxyapatite with cerium and fluoride ions by replacing calcium and hydroxide moieties. In the right part of the diagram in Figure 6C, a protective mechanism is suggested by sequential rinsing with cerium and fluoride solutions in the case of caries. 

When the integrity of the enamel is violated, dentin (consisting mainly of collagens) channels are opened. Moreover, carious lesions contain numerous surface defects that are colonized by bacteria and covered with biofilms. Cerium ions are readily adsorbed by collagens, polysaccharides and other biopolymers. Then, during treatment with fluoride ions, a protective layer consisting of cerium fluoride nanoparticles is formed in situ on the surface of the caries zone in the saliva environment. If a certain amount of terbium is added to the solution of cerium ions (as a label), this layer of nanoparticles becomes visible, due to the activated luminescence, which is shown in Figure 6B. At the same time, the mechanism of enamel strengthening in the healthy part of the carious tooth also takes place as can be seen in diagram Figure 6C (left).

### 3.4. Toxicity Analysis of the Effect of Cerium Fluoride Nanoparticles on Dental Pulp Stem Cells (DPSc)

**Figure 8:**
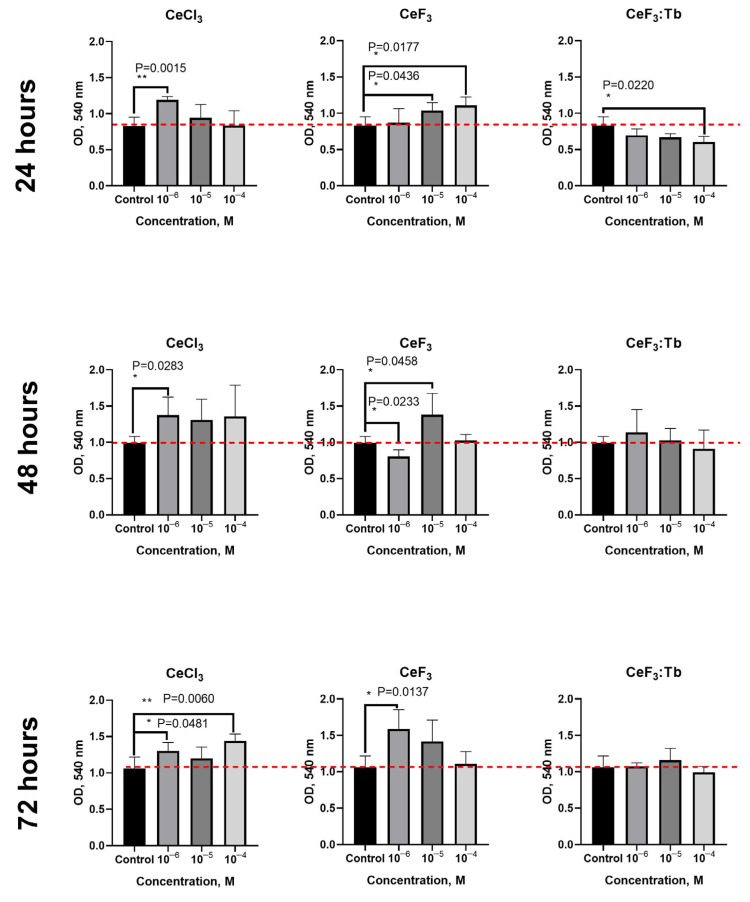
Metabolic activity (via MTT assay) of DPSc in the presence of CeCl_3_, CeF_3_ and CeF_3_:Tb nanoparticles (10^−4^–10^−6^ M) after 24, 48 and 72 h of incubation. The data are presented as the mean ± SD. * Significant differences were assessed using the Mann–Whitney U criterion.

After treatment of the oral cavity with fluoride and cerium ions, cerium fluoride nanoparticles can deposit on the mucous membranes and dental surfaces and can remain there for a long time. Therefore, it was decided to study the biological behaviour of such nanoparticles on dental pulp stem cells (DPSc) in more detail. First, the cytotoxicity of CeF_3_ and CeF_3_:Tb nanoparticles was analysed on DPSc culture after 24, 48 and 72 h of cultivation (Figure 8). Additionally, cerium chloride was used as a reference for the comparative analysis of cerium-containing nanoparticles’ bioactivity. It was observed that cerium chloride at a concentration of 10^–6^ M caused a significant increase in the optical density of formazan, which confirmed the increased level of metabolic activity of the DPSc. 

CeF_3_ nanoparticles also increased the level of metabolic activity of cell culture at 10^−4^ M (*p* ≤ 0.0177) and 10^−5^ M (*p* ≤ 0.0436) concentrations, compared with the untreated control group. In turn, CeF_3_:Tb nanoparticles throughout the concentration range studied did not significantly increase the level of metabolic activity of the cells after 24 h of cultivation. After 48 h, a similar effect was observed for CeF_3_ nanoparticles and cerium chloride. After 72 h, CeF_3_ nanoparticles at a concentration of 10^−6^ M significantly increased (by ~30%) the level of metabolic activity of DPSc, while CeF_3_:Tb did not show similar biological activity. An increase in human mesenchymal stem cells proliferation rate by cerium oxide nanoparticles has already been shown [29,30], whereas the positive effect of cerium fluoride nanoparticles has not been observed previously.

**Figure 9:**
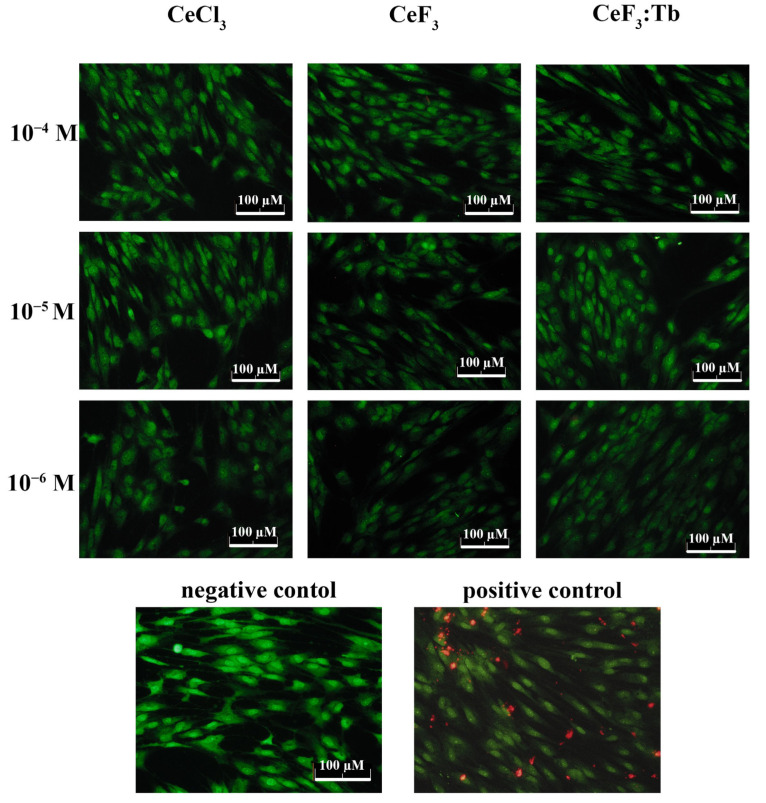
Live/Dead assay for DPSc after 24 h incubation with different concentrations (10^−4^–10^−6^ M) of CeCl_3_ solution, CeF_3_ and CeF_3_:Tb nanoparticles. Negative control—cells with no nanoparticles. Positive control—1 mM H_2_O_2_ for 1 h.

Next, an analysis was made of the viability of DPSc after incubation with CeF_3_ and CeF_3_:Tb nanoparticles (10^−4^–10^−6^ M) using a Live/Dead Viability Kit (Figure 9). Treatment with hydrogen peroxide (1 mM H_2_O_2_ for 1 h) was used as a positive control (100% death). None of the investigated concentrations of CeF_3_ and CeF_3_:Tb nanoparticles caused the appearance of dead (positively stained with propidium iodide) cells after 24 h of incubation. The morphological characteristics of the cells did not change after incubation with CeF_3_ and CeF_3_:Tb nanoparticles; DPSc retained their elongated shape, characterizing active proliferation and migration. Even the highest concentrations of CeF_3_ and CeF_3_:Tb nanoparticles (10^−4^ M) caused neither cell death nor changes in the cells’ morphology.

**Figure 10:**
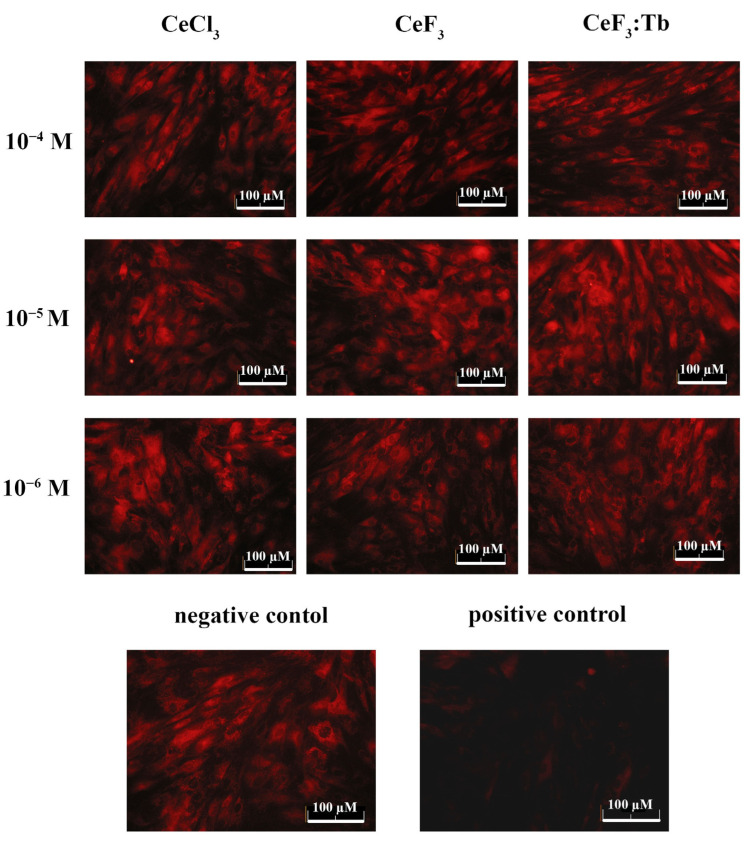
Analysis of the membrane mitochondrial potential of DPSc after 24 h of incubation with different concentrations (10^−4^–10^−6^ M) of CeCl_3_ solution, CeF_3_ and CeF_3_:Tb nanoparticles. Negative control—cells without nanoparticles. Positive control—valinomycin 1 µM.

Considering that rare earth fluoride nanoparticles exhibit notable redox activity [31] and are able to modulate the redox status of a cell, we decided to measure the level of membrane mitochondrial potential (MMP), which is an indicator of the metabolic activity of cells (Figure 10) and is significantly reduced upon the development of intracellular oxidative stress. Using the potential-sensitive dye TRME, the mitochondrial potential level was analysed after 24 h of incubation with CeF_3_ and CeF_3_:Tb nanoparticles (10^−4^–10^−6^ M). Valinomycin (1 μM) was used as a positive control. This substance is a depolarizing agent that significantly reduces the MMP level. None of the tested concentrations of cerium fluoride nanoparticles and cerium chloride solutions (10^−4^–10^−6^ M) caused a decrease in the MMP level.

**Figure 11:**
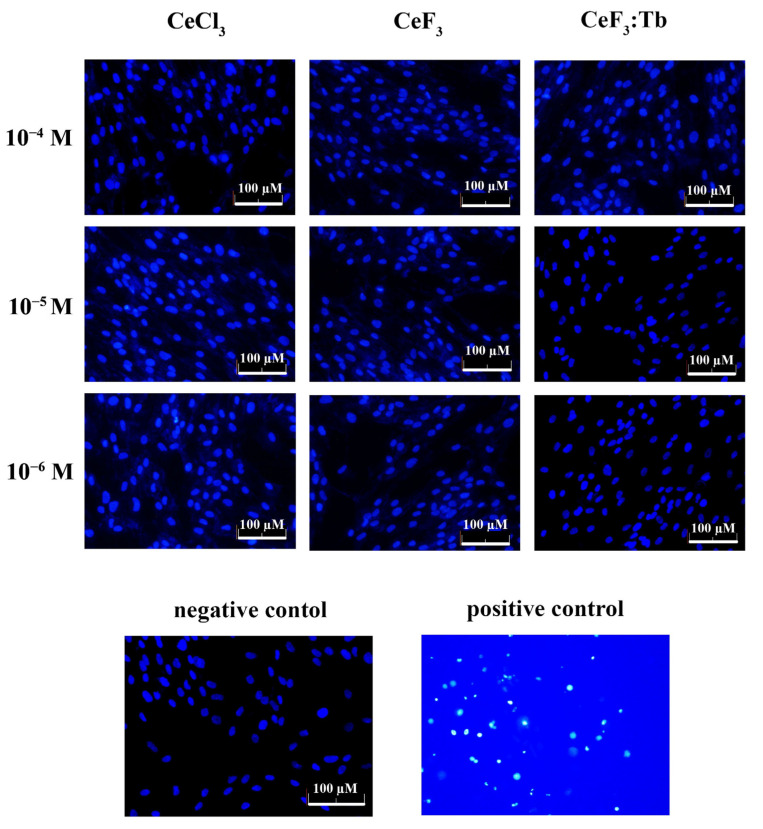
Fluorescent microscopy analysis of nuclei fragmentation in DPSc after 24 h incubation with different concentrations (10^–4^–10^–6^ M) of CeCl_3_ solution, CeF_3_ and CeF_3_:Tb nanoparticles, using Hoechst 33342. Negative control—cells without nanoparticles. Positive control—10 min UVB irradiation.

The genotoxicity analysis of cerium fluoride and terbium-doped cerium fluoride nanoparticles was performed by cell staining with Hoechst 33342 (Figure 11). As a positive control, UV-irradiation (365 nm, 10 min) was used, causing a significant increase in the number of dead cells, as observed in chromatin condensation or its defragmentation. None of the investigated concentrations of cerium fluoride and terbium-doped cerium fluoride nanoparticles caused any morphological changes in the nuclear apparatus of human mesenchymal stem cells.

**Figure 12:**
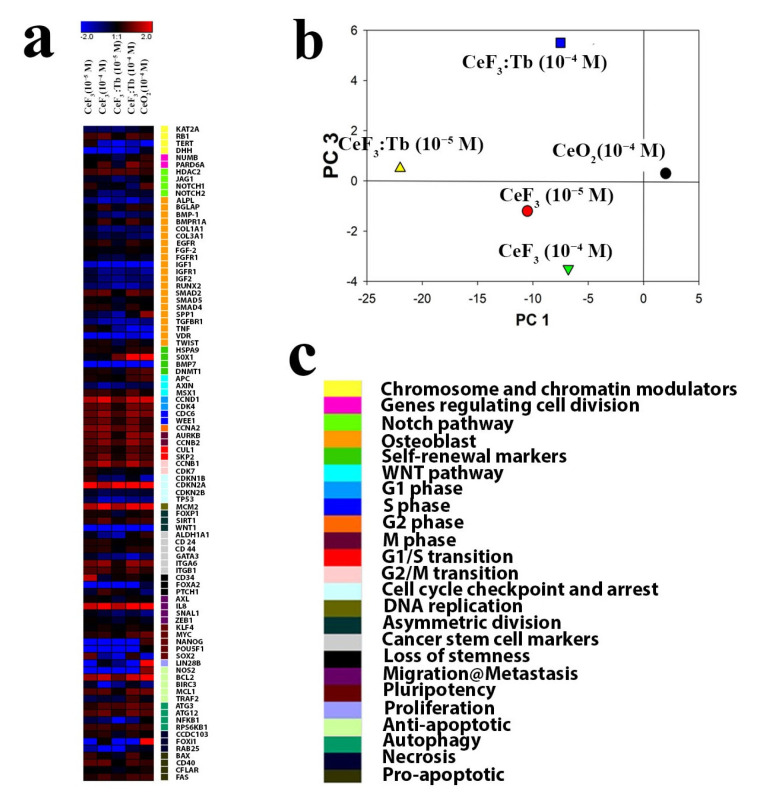
Heat map of gene expression in DPSc after 24 h incubation with CeF_3_ (10^−4^ and 10^−5^ M) and CeF_3_:Tb (10^−4^ and 10^−5^ M) nanoparticles (**a**). The intensity scale of the standardized expression values ranges from −3 (green: low expression) to +3 (red: high expression), with a 1:1 intensity value (black) representing the control (nontreated). Principal Component Analysis (PCA) of qRT-PCR data for different concentrations of CeF_3_ and CeF_3_:Tb nanoparticles (**b**). Cluster groups of genes and their functionality (**c**).

Next, a comprehensive analysis was performed regarding the gene expression (more than 90 genes) responsible for proliferation, differentiation, pluripotency, migration, apoptosis and other important cellular functions after incubation with CeF_3_ and CeF_3_:Tb nanoparticles (Figure 12). It should be noted that both types of nanoparticle significantly increased the expression of genes responsible for the cell cycle, differentiation and proliferation. These data make it possible to confirm the stimulating effect of cerium fluoride nanoparticles on the proliferation of human DPSc. 

The authors have previously shown that citrate-stabilized cerium oxide nanoparticles increase the proliferation rate of human MSCs isolated from dental pulp [32] and human vartan jelly [33]. This stimulation was associated with the regulation of the redox status of DPSc and activating genes responsible for proliferative and migratory activity. CeF_3_:Tb nanoparticles (10^−4^ M) increased mRNA transcripts of SOX2, a stem cell marker gene, which is required to maintain the self-renewal or pluripotency of mesenchymal stem cells [34]. Interestingly, all types of tested nanoparticles activated cell proliferation marker genes (CCDN1, CDK4, CDC6, WEE1, CCNA2, AURKB, CCNB2, CUL1, SKP2, CCNB1 and CDKN2A), with a concentration dependence observed. 

CeF_3_ and CeF_3_:Tb nanoparticles at a 10^−4^ M concentration led to the activation of far more genes than the lower concentration (10^−5^ M) of these nanoparticles. We note the overexpression of the MCM2 gene, which encodes the DNA replication licensing factor MCM2 and which is a DNA replication marker [35]. This MCM2 overexpression indirectly confirms the high replicative activity of human DPSc in the presence of cerium fluoride nanoparticles. All investigated concentrations upregulate the IL-8 gene, which is responsible for cell migration, and the BCL2 gene, a marker of anti-apoptotic activity. 

A comparison of different concentrations of CeF_3_ nanoparticles by PCA analysis revealed a similar trend in the gene activation for both concentrations (10^−4^ and 10^−5^ M), while CeF_3_:Tb showed a dose-dependent activation (Figure 12b). These results indicate that the doping of cerium fluoride nanoparticles changes their biological activity, which is reflected in the level of gene expression. 

In general, based on data on transcriptional activity, it can be concluded that both CeF_3_ and CeF_3_:Tb nanoparticles did not activate the osteogenic differentiation of stem cells but promoted their proliferation and migration in a dose-dependent manner. At the same time, there was no activation of genes responsible for apoptosis, inflammation and markers of cell death; in fact, on the contrary, there was a pronounced expression of the gene encoding the anti-apoptotic protein BCL2.

## 4. Discussion

A comprehensive analysis was performed of cerium and calcium species’ action on epithelial cells and interaction with such toxic compounds as ammonium fluoride or hydrofluoric acid. The results obtained revealed that the pre-treatment of epithelial cells with cerium chloride solutions at a high concentration led to a significant inhibition of their metabolic activity; however, in the case of their subsequent treatment with an equimolar concentration of ammonium fluoride, this toxic effect levelled. At the same time, pre-treatment of epithelial cells with a solution of calcium chloride did not provide such a protective effect. 

This protective effect was also absent in the case of the pre-treatment of cells with cerium oxide nanoparticles. These data confirm the in situ formation of cerium fluoride nanoparticles upon sequential treatment with water soluble cerium and fluoride compounds. Considering that the antioxidant properties of various cerium compounds have been well studied recently, an analysis was made of the possible protective effect of such a pre-treatment of epithelial cells in a model of oxidative stress induced by hydrogen peroxide. 

Pre-treatment of the cells, even with micromolar concentrations of cerium chloride, resulted in a pronounced antioxidant effect, in contrast to calcium compounds, which did not exhibit any protective activity at similar concentrations. Cerium fluoride nanoparticles demonstrated an even more pronounced protective effect than cerium ions, which confirms the promise of using this nanomaterial as an effective protective agent with high antioxidant activity.

At present, the problem of the development of carious lesions of tooth enamel remains unresolved, due to the influence of bacterial glycolysis products and the subsequent dissolution of calcium phosphates of tooth enamel. The proposed sequential scheme of teeth treatment with solutions of cerium chloride and ammonium fluoride significantly inhibits acid corrosion of tooth enamel, preventing calcium leaching. 

When a tooth with damaged enamel is subjected to successive treatment with aqueous solutions of cerium and fluoride species, a layer of cerium fluoride nanoparticles is formed in the carious zone, which acts as a protective layer that prevents the processes of enamel destruction. In turn, the formation of luminescent terbium-doped cerium fluoride nanoparticles upon sequential teeth treatment with a mixture of cerium and terbium species and fluoride species enables the visualization of tooth enamel microcracks. 

A comprehensive analysis of the biological activity of cerium fluoride nanoparticles showed that they can act as a mitogen, accelerating the proliferation of human dental cells, as well as activating the key signalling pathways of cells responsible for the proliferation, differentiation and redox status of human MSCs. These data are in good agreement with the authors’ previous report that demonstrated that cerium fluoride nanoparticles can act as mitogens for whole organisms [8]. 

The downregulation of more than 30 genes responsible for cell differentiation and redox status was shown in the presence of CeF_3_ and CeF_3_:Tb nanoparticles, which was not observed in the case of treatment with cerium oxide nanoparticles at the same concentration. At the same time, an increase in the expression of markers of apoptosis and inflammation was not observed at all, which confirms the high level of biocompatibility of nanoscale cerium fluoride and cerium oxide.

In the context of practical application, the proposed teeth treatment method has some issues that must be taken into account when considering future in vivo studies. The use of oral rinses inevitably results in them being swallowed. Most contemporary dentrifices contain fluorides, and so the risk of their ingestion has been thoroughly assessed [36,37]. On the other hand, the use of rare earth compounds in oral rinses has received barely any attention. Cerium species, however, have a long history of biomedical tests [9]. They have relatively low toxicity per os: LD_50_ 4200 mg kg^−1^ for cerium nitrate (female rats) and LD_50_ 1178 mg kg^−1^ (female mice) [38]. These values are close to the toxicity parameters of table salt (LD_50_ 3000 mg kg^−1^ for rats [39]). 

The low risk associated with the oral administration of cerium species is supported by their low absorption, with more than 95% of the species being excreted in the faeces [9,40]. Moreover, orally administered lanthanide compounds at low doses have even been reported to have beneficial effects for laboratory and farm animals [9,41] resulting in a significant increase in their immune level [9]. According to the most recent report, orally administered cerium species have great potential for use in dental practise as they prevent inflammatory changes in periodontal tissues in obese humans with generalized catarrhal gingivitis [42].

Further implementation of the current research requires the translation of in vitro experiments to preclinical and clinical trials, as well as conversion of the cellular half maximal inhibitory concentration IC_50_ (mmol/L) into corresponding toxicity values for animals and humans (mg/kg), such as median lethal dose LD_50_, no observed adverse effect level NOAEL and no observed effect level NOEL. According to a prediction model recommended by the International Workshop on In Vitro Methods for Assessing Acute Systemic Toxicity [43], LD_50_ values (in mmol/kg) can be estimated from IC_50x_ values (in mmol/L) using the following relation: log (LD_50_) = 0.435 × log (IC_50x_) + 0.625.

For cerium (III) chloride, IC_50_ = 5–10 mM (see above), thus LD_50_ is estimated to be 8.5 –11.5 mmol/kg or 2100–2800 mg/kg, which is in good agreement with experimentally measured values of 2800 mg/kg (male and female rats per os) [44]. NOEL and NOAEL are also assessed prior to the initiation of human trials, in order to establish a safe clinical starting dose. For cerium nitrate, NOEL was reported to be 110 mg/kg/day and NOAEL was reported to be 330 mg/kg/day (47-day, rats per os) [44]. 

For a more accurate transfer of drug doses from animal to human trials, the body surface area (BSA) normalization method has been suggested [45]. BSA normalization was advocated for translation from animals to humans in phase I and phase II clinical trials: $\mathrm{HED}=\mathrm{Animal}\;\mathrm{dose}\times \frac{\mathrm{Animal}\;\mathrm{Km}}{\mathrm{Human}\;\mathrm{Km}}$ (here, HED—human equivalent dose (mg/kg), Km—surface area factor). For rats, Km = 6 and, for humans, Km = 37 (adults) or 25 (children) [45].

Taking into account the above considerations, the NOEL value for water-soluble cerium species for adult humans can be estimated as 18 mg/kg/day or about 1 g/day (for the body weight ~60 kg). Common oral rinses contain ~0.05% (230 ppm F) NaF, and thus the accidental daily swallowing of 10 mL rinse solution is equal to the ingestion of ~2.3 mg of fluoride [46], while the probable toxic dose (PTD) of fluoride was estimated to be 5 mg/kg [36]. 

According to the principle of molar quantitative compliance (see above), the cerium-containing rinse solution should contain approximately 0.1% CeCl_3_, so that accidental daily swallowing of 10 mL rinse solution will result in the ingestion of only ~10 mg of CeCl_3_, which is an order of magnitude less than the estimated NOEL value. This confirms the suitability of the proposed protocol of two-component oral treatment for future clinical trials.

## 5. Conclusions

The sequential use of CeCl_3_ and NH_4_F solutions in equimolar sub-toxic concentrations enables avoidance of the possible toxic effects of each substance, ensuring the preservation of the metabolic activity of the cells. Cerium fluoride nanoparticles and terbium-doped cerium fluoride nanoparticles do not exhibit cytotoxicity and genotoxicity to DPSc, even at high concentrations (10^−4^ M). 

Cerium fluoride nanoparticles in millimolar concentrations (from 10^−5^–10^−6^ M) are able to accelerate cell proliferation rate after 72 h of incubation, maintaining a high level of metabolic activity and mitochondrial potential of the cell culture. CeF_3_ and CeF_3_:Tb nanoparticles significantly increase the expression of genes responsible for the cell cycle, differentiation and proliferation. These findings make it possible to confirm the stimulating effect of cerium fluoride nanoparticles on the proliferation of human DPSc.

The formation of cerium fluoride on the surface of the mucous membrane and teeth can protect them from the development of carious lesions, periodontitis, ROS attacks and other inflammatory diseases of the oral cavity.

## Figures and Tables

**Table 1:** Selected genes and their functions.

Gene	Function	Gene	Function	Gene	Function
KAT2A	Chromatin and chromosome modulators	HSPA9	Self-renewal markers	FOXA2	Stemness reduction markers
RB1		SOX1		PTCH1	
TERT		BMP7		CD34	
		DNMT1			
DHH	Regulators of cell division symmetry			AXL	Cell migration markers
NUMB		APC	Stemness maintenance (Wnt signalling)	IL8	
PARD6A		AXIN		SNAI1	
HDAC2	Stemness maintenance (Notch signalling)	MSX1		TWIST1	
JAG1		CCND1	Proliferation markers	ZEB1	
NOTCH1		CDK4		KLF4	Pluripotency markers
NOTCH2		CDC6		MYC	
ALPL		WEE1		NANOG	
BGLAP	Markers of MSCs and their differentiation	CCNA2		POU5F1	
BMP1		AURKB		SOX2	
BMPR1A		CCNB2		NOS2	Anti-apoptotic markers
COL1A1		CUL1		BCL2	
COL3A1		SKP2		BIRC3	
EGFR		CCNB1		MCL1	
FGF-2		CDK7		TRAF2	
FGFR1		CDKN1B		ATG3	Autophagy markers
IGF1		CDKN2A		ATG12	
IGFR1		CDKN2B		NFKB1	
RUNX2		TP53		RPS6KB1	
SMAD2		MCM2		CCDC103	Necrosis markers
SMAD4		LIN28B		FOXI1	
SMAD5		FOXP1	Markers of asymmetric cell division	JPH3	
SPP1		SIRT1		RAB25	
TGFBR1		WNT1		BAX	Pro-apoptotic markers
TNF		ALDH1A	Stemness markers	CD40	
VDR		CD44		CFLAR	
		GATA3		FAS	
		ITGA6		TNFRSF10A	
		ITGB1			

## Data Availability

The data are included within the article.
